# Cultural Determinants of Chronic Disease Management: A Cross-Comparative Medical Review

**DOI:** 10.3390/healthcare14050640

**Published:** 2026-03-03

**Authors:** Ismihan Almasa Uddin, Rafay Mujahid Siddiqui

**Affiliations:** Chicago College of Osteopathic Medicine, Midwestern University, Downers Grove, IL 60515, USA; rafay.siddiqui1@midwestern.edu

**Keywords:** sociocultural determinants, chronic disease management, cross-cultural health disparities, culturally tailored interventions

## Abstract

Chronic diseases—including diabetes mellitus, cardiovascular disease, chronic kidney disease, and autoimmune disorders—remain the leading causes of global morbidity and mortality. While biomedical pathophysiology defines the etiology and progression of these conditions, cultural factors significantly modulate how patients perceive illness, engage in treatment, and adhere to medical recommendations. This review synthesizes evidence from cross-cultural studies, with a specific focus on medical manifestations and therapeutic challenges, to examine how sociocultural determinants intersect with biological disease processes. We highlight nuanced case comparisons between South Asian, East Asian, Middle Eastern, African, Latinx, and Indigenous populations, illustrating how cultural constructs such as collectivism, fatalism, stigma, reliance on traditional medicine, and health literacy directly influence outcomes in chronic disease management. Importantly, we integrate evidence-based recommendations for healthcare professionals, emphasizing culturally tailored interventions, precision medicine approaches, and the role of interdisciplinary care teams.

## 1. Introduction

**The Global Burden of Chronic Disease**

Chronic non-communicable diseases (CNCDs) account for more than 70% of deaths worldwide, with cardiovascular disease alone responsible for an estimated 17.9 million annual deaths, followed by malignancies, chronic respiratory diseases, and diabetes mellitus [1,2,3]. While genetic predisposition, diet, and environmental exposures serve as universal contributors, patient engagement with healthcare systems is profoundly shaped by sociocultural contexts. For clinicians, cultural competence is no longer ancillary but central to chronic disease care.

**Culture as a Medical Determinant**

Culture, operationally defined as shared values, beliefs, language, and health practices within a community, exerts physiological and clinical effects on chronic disease outcomes. This is not simply a matter of “patient preference”. For example, delayed initiation of insulin in South Asian populations is frequently linked to stigma surrounding injectable therapy, yet such delay demonstrably increases the risk of diabetic retinopathy and nephropathy [4]. Similarly, East Asian patients with hypertension often exhibit lower medication preference when traditional remedies (e.g., Kampo or Traditional Chinese Medicine formulations) are prioritized over guideline-directed antihypertensives [5]. These examples illustrate that cultural variables must be investigated with the same rigor as laboratory findings.

**Cross-Cultural Variability in Chronic Disease Presentation**

**A Comparative Analysis Across Cultures Demonstrates Distinct Disease Burdens and Presentations:**

- South Asians: Elevated rates of premature coronary artery disease, linked not only to metabolic syndrome but also cultural dietary practices emphasizing refined carbohydrates and ghee-rich foods [6].
- Middle Eastern populations: High prevalence of type 2 diabetes, worsened during Ramadan fasting periods when dietary patterns shift toward carbohydrate-dense night-time meals [7].
- African and Afro-diasporic groups: Increased incidence of hypertensive nephrosclerosis, often exacerbated by mistrust of medical systems [8].
- Latinx populations: Greater risk of poorly controlled asthma, driven by environmental and psychosocial factors; family dynamics can complicate controller adherence in Latino adolescents [9]
- Indigenous populations: High rates of systemic lupus erythematosus, combined with ethnic disparities in disease expression and outcomes and limited access to rheumatology care, lead to higher disease activity, accelerated damage, and a poorer overall prognosis [10].

Each of these patterns demonstrates that cultural frameworks intersect directly with disease-specific pathophysiology, complicating management strategies if left unaddressed.

**Beyond Sociological Framing: A Medical Imperative**

Although culture is often approached through the lens of anthropology or sociology, this review adopts a strictly medical orientation. We analyze culture as a clinical risk factor that modifies disease trajectories at the levels of:

- Pathophysiology: e.g., Ramadan-related circadian misalignment in diabetic patients leading to impaired β-cell recovery.
- Treatment adherence: e.g., cultural fatalism in some Latinx groups undermining participation in chemotherapeutic regimens.
- Healthcare utilization: e.g., reliance on traditional healers delaying emergency cardiovascular interventions.

This review will therefore situate cultural differences not as external modifiers but as intrinsic variables influencing morbidity and mortality.

**Role of Healthcare Professionals**

Healthcare professionals must act not only as diagnosticians and prescribers but also as cultural interpreters. Culturally incongruent care leads to poorer outcomes and a marked inability to provide a higher standard of care [11]. Training programs in “cultural humility” and the integration of medical anthropologists into clinical care teams are emerging but remain underutilized. Moreover, while cultural competence training often remains general and superficial, this review argues for disease-specific cultural tailoring, as in the following examples:

- In endocrinology: anticipatory insulin education for South Asian patients resistant to initiation.
- In cardiology: culturally framed sodium-reduction strategies in East Asian populations reliant on fermented soy products.
- In oncology: integration of curandero figures in Latinx cancer care pathways to increase chemotherapy adherence.

**Objectives of This Review**

The primary objective of this article is to provide healthcare professionals with an in-depth, medically grounded synthesis of cultural determinants of chronic disease care. Specifically, we will:

1. Analyze pathophysiological and cultural intersections in chronic disease progression.
2. Provide detailed cross-cultural case comparisons with clinical relevance.
3. Identify evidence-based strategies which healthcare professionals can adopt for culturally aligned interventions.
4. Offer forward-looking recommendations for integrating culture into precision medicine frameworks.

In this way, culture is redefined not as a barrier to care but as a therapeutic variable—one which clinicians can leverage to optimize chronic disease outcomes globally.

## 2. Pathophysiology Meets Culture: Disease-Specific Perspectives

**A.**  **Diabetes Mellitus**

**Biomedical Pathophysiology**

Type 2 diabetes mellitus (T2DM) arises from insulin resistance in the hepatic and peripheral (skeletal muscle and adipose) tissues, combined with β-cell dysfunction leading to relative insulin deficiency. Chronic hyperglycemia accelerates non-enzymatic glycation of proteins, promotes oxidative stress, and damages microvascular and macrovascular beds, yielding complications such as diabetic nephropathy, retinopathy, neuropathy, and accelerated atherosclerosis [12].

**Cultural Influences on Disease Course**

**South Asian Populations**

South Asians develop T2DM at lower BMI thresholds and younger ages due to intrinsic β-cell fragility and visceral adiposity. However, cultural dietary norms—such as high reliance on polished rice, refined wheat flour (maida), and clarified butter (ghee)—exacerbate hyperinsulinemia and postprandial glucose spikes. Furthermore, cultural stigma surrounding insulin injections results in delayed therapy escalation, directly raising the incidence of proliferative retinopathy and albuminuria [4].

**Middle Eastern Populations**

During Ramadan fasting, circadian misalignment and nocturnal hypercaloric intake lead to greater glycemic variability. Case series demonstrate increased incidence of diabetic ketoacidosis (DKA) in young adults and severe hypoglycemia in insulin-treated patients during Ramadan [7]. Endocrinologists must anticipate this cultural context, proactively adjusting insulin regimens (e.g., switching from basal-bolus to basal-plus with nocturnal correction doses).

**East Asian Populations**

Patients often integrate Traditional Chinese Medicine (TCM), such as bitter melon (Momordica charantia) or berberine supplements, which may lower HbA1c modestly [13] but risk herb–drug interactions with metformin or sulfonylureas. Over-reliance delays the use of evidence-based incretin therapy (GLP-1 receptor agonists, DPP-4 inhibitors).

**B.**  **Cardiovascular Disease (Cvd)**

**Biomedical Pathophysiology**

Atherosclerosis is driven by endothelial dysfunction, LDL oxidation, macrophage foam cell formation, and plaque instability. Acute coronary syndromes occur when rupture or erosion leads to thrombus formation. Hypertension and left ventricular hypertrophy accelerate heart failure progression.

**Cultural Influences on Disease Course**

**South Asians**

Premature coronary artery disease (CAD) is markedly more prevalent, with high rates of multi-vessel disease in individuals under 50. Beyond genetics (e.g., elevated lipoprotein(a)), cultural dietary sodium and carbohydrate excess contribute to metabolic syndrome. Physical inactivity, particularly in urban diaspora populations, compounds risks [6].

**East Asians**

Stroke predominates over myocardial infarction as the cardiovascular endpoint; this is related to a higher prevalence of hemorrhagic stroke. Hypertension often goes undertreated when patients substitute antihypertensives with herbal decoctions like Tian Ma (Gastrodia elata), leading to poor blood pressure control [5].

**African and Afro-Diasporic Groups**

Hypertensive heart disease and hypertensive nephrosclerosis are disproportionately common. Beyond APOL1 polymorphisms, cultural mistrust of the healthcare system contributes to low medication adherence. Delays in seeking care for angina or heart failure exacerbate disease severity at presentation [14].

**C.**  **Chronic Kidney Disease (Ckd)**

**Biomedical Pathophysiology**

CKD arises from progressive nephron loss due to diabetes, hypertension, glomerulonephritides, or genetic mutations. Hyperfiltration injury, glomerulosclerosis, and tubulointerstitial fibrosis contribute to renal decline. Uremia induces cardiovascular mortality via vascular calcification, oxidative stress, and systemic inflammation [15].

**Cultural Influences on Disease Course**

**African Descent**

APOL1 risk alleles explain some predisposition, but traditional medicine often compounds nephrotoxicity. Herbal remedies containing aristolochic acid or heavy metals accelerate tubulointerstitial damage, hastening ESRD progression [16].

**Latinx Populations**

Chronic kidney disease of unknown origin (CKDu), particularly among Central American agricultural workers, is exacerbated by cultural acceptance of grueling labor in high-heat conditions without hydration. A culturally embedded work ethic deters reporting of symptoms until late stages, leading to advanced ESRD presentations requiring emergent dialysis initiation [17].

**Indigenous Populations**

Limited access to renal replacement therapy intersects with cultural resistance to hemodialysis, viewed in some communities as invasive or spiritually incongruent. Care access barriers and historical factors contribute to lower uptake of renal replacement therapies; culturally safe, community-engaged approaches are recommended [18].

**D.**  **Autoimmune Disorders**

**Biomedical Pathophysiology**

Autoimmune diseases, including systemic lupus erythematosus (SLE), rheumatoid arthritis, and multiple sclerosis, arise from dysregulated adaptive immunity. Loss of tolerance permits autoreactive B and T cells, while cytokines such as IL-6, TNF-α, and IFN-α drive tissue inflammation.

**Cultural Influences on Disease Course**

**Indigenous Populations**

Indigenous North American women have some of the highest global prevalence rates of SLE, presenting with aggressive renal involvement (lupus nephritis class IV). Limited access to rheumatologists, compounded by cultural reliance on traditional healers, leads to diagnostic delay and greater incidence of progression to ESRD [10].

**East Asian Populations**

Autoimmune thyroiditis (Hashimoto’s, Graves’) often coincides with psychiatric symptoms [19], leading to misattribution of thyrotoxicosis-related anxiety and delayed endocrinology referral.

## 3. Case Comparisons Across Cultures

To underscore the intersection between molecular pathophysiology and sociocultural determinants, this section presents four detailed case vignettes, each anchored in peer-reviewed evidence, illustrating culturally driven variations in presentation, adherence, and outcome of chronic disease.

**Case 1: Ramadan and Insulin Adjustment in Type 2 Diabetes Mellitus**

**Vignette**

A 45-year-old Egyptian man with 12 years of poorly controlled type 2 diabetes mellitus (HbA1c 9.6%) presents during Ramadan. His baseline regimen includes basal-bolus insulin (glargine 30 units nocte, lispro 8 units with each meal). He fasts from dawn to sunset, consuming high-carbohydrate meals after sunset (iftar) and before dawn (suhoor). Within 10 days of fasting, he experiences three hypoglycemic episodes during mid-afternoon and one episode of postprandial hyperglycemia exceeding 400 mg/dL after iftar.

**Biomedical Considerations**

Circadian insulin sensitivity declines with nocturnal feeding.

Counter-regulatory hormones (cortisol, growth hormone) surge in the early morning, elevating fasting glucose.

Missed daytime meals decrease prandial insulin demand, while nocturnal hypercaloric meals increase bolus requirements.

**Cultural Context**

The patient views fasting as non-negotiable, tied to spiritual identity. His reluctance to “break fast” for hypoglycemia leads to risk of seizures or arrhythmias.

**Clinical Approach**

Guidelines recommend pre-Ramadan counseling, reducing dose of basal insulin by 15–30%, and transitioning to basal-plus regimens. Pre-Ramadan plans should emphasize individualized reductions in insulin dose along with close glucose monitoring, consistent with established Ramadan diabetes guidance [7]. The endocrinologist balances biomedical risk with cultural sensitivity, framing adjustments as enabling rather than prohibiting religious practice.

**Case 2: Lupus Nephritis in Indigenous Populations**

**Vignette**

A 27-year-old Cree woman presents with facial edema, arthralgias, and hematuria. Workup reveals ANA positivity (1:1280), anti-dsDNA positivity, low complement levels, and nephrotic-range proteinuria. Renal biopsy demonstrates class IV lupus nephritis. The patient delayed seeking care, initially pursuing traditional healing practices (sweat lodges, herbal infusions) for six months before hospital admission.

**Biomedical Considerations**

Class IV lupus nephritis carries high risk of progression to end-stage renal disease (ESRD).

Early immunosuppression with corticosteroids plus mycophenolate mofetil or cyclophosphamide improves renal survival.

Delay accelerates glomerulosclerosis and interstitial fibrosis, reducing therapeutic reversibility.

**Cultural Context**

The patient reports distrust of urban hospitals, preferring community healers. Family consensus and cultural rituals play a central role in treatment decisions. Dialysis is perceived as “unnatural” disruption of bodily integrity.

**Clinical Approach**

Nephrology teams partner with community health representatives fluent in Cree language. Treatment framing emphasizes corticosteroids as “resetting the body’s defenses” rather than “suppressing” immunity, aligning with Indigenous concepts of restoring balance. Shared decision-making integrates traditional healers alongside rheumatologists, improving adherence.

**Case 3: Chronic Kidney Disease in Central American Agricultural Workers**

**Vignette**

A 38-year-old male sugarcane worker from El Salvador presents with fatigue, flank pain, and recurrent muscle cramps. Laboratory evaluation reveals serum creatinine 2.8 mg/dL, eGFR 26 mL/min/1.73 m^2^, and minimal proteinuria. He denies diabetes or hypertension.

**Biomedical Considerations**

This pattern corresponds to Mesoamerican nephropathy (CKDu), characterized by tubulointerstitial injury, minimal proteinuria, and progressive renal failure. Pathogenesis is hypothesized to involve recurrent heat stress, volume depletion, hyperuricemia, and nephrotoxic agrochemical exposure [17].

**Cultural Context**

Workers pride themselves on “aguantar” (enduring) heat and labor. Breaks or hydration are culturally stigmatized as weakness. Seeking care threatens employment stability.

**Clinical Approach**

- Primary prevention focuses on hydration stations and shaded rest, but uptake requires cultural re-framing (emphasizing collective strength rather than individual weakness).
- Pharmacologic interventions are limited; ACE inhibitors or uric-acid-lowering therapy offer uncertain benefit.
- Community-based participatory research involving campesino leaders enhances trust and facilitates acceptance of screening programs.

**Case 4: Hypertension and Stroke in East Asian Populations**

**Vignette**

A 64-year-old Japanese woman with known hypertension presents with acute-onset left-sided hemiparesis. Blood pressure is 220/120 mmHg. CT brain reveals a large right basal ganglia intracerebral hemorrhage. Her family reports she had been taking Kampo herbal preparations (Tian Ma) for blood pressure control rather than prescribed calcium-channel blockers.

**Biomedical Considerations**

East Asian populations exhibit higher stroke-to-MI ratios compared to Western cohorts.

Intracerebral hemorrhage is particularly common due to higher prevalence of cerebral small-vessel disease and poor BP control.

Delay in initiation of antihypertensives results in fragile lenticulostriate arterioles prone to rupture.

**Cultural Context**

Family members expressed belief that Western medicines “weaken kidneys” and “accumulate toxins”, while herbal preparations are “natural” and safer. The patient distrusted repeated monitoring visits.

**Clinical Approach**

Neurologists and internists must counsel patients with cultural humility, acknowledging the legitimacy of Kampo within historical Japanese medicine, while providing clear data on the stroke prevention benefits of pharmacologic BP control. Collaborative integrative clinics where Kampo practitioners work alongside internists have demonstrated improved adherence to antihypertensives [5].

## 4. Future Directions in Culturally Responsive Chronic Disease Management

The preceding sections demonstrate that chronic diseases—though universal in their biological substrate—are experienced, expressed, and managed through culturally inflected pathways. Moving forward, the task for clinicians, researchers, and health systems is to translate transcultural insights into structured medical frameworks that can be consistently applied in both high-resource and resource-limited contexts. Part 4 highlights three central domains: (1) translational research in transcultural medicine, (2) integration into clinical training and practice, and (3) structural and policy-level considerations to ensure equitable access.

## 5. Translational Research in Transcultural Medicine

A. Pharmacogenomic integration.

Although pharmacogenomic screening is becoming standard in oncology and cardiology in high-income countries, its integration remains inconsistent globally. Expanding CYP2C19 genotyping for East Asian cardiovascular patients [20], DPD testing in fluoropyrimidine-based chemotherapy, and APOL1 screening in nephrology [21] could all reduce adverse outcomes. However, cultural considerations must be acknowledged: some Indigenous communities resist genetic testing due to historical exploitation in research [22]. Future research should prioritize community-driven frameworks to build trust while ensuring clinical utility.

B. Metabolomic and immunologic profiling.

Advances in metabolomics reveal population-specific biomarkers that predict risk of chronic disease. For example, genetic variants in APOL1 are a major contributor to the increased risk of kidney disease in people of recent African ancestry [21]. Longitudinal cross-cultural studies are urgently needed to identify these pathways and inform therapeutic decision-making.

C. Clinical trials with cultural heterogeneity.

Current clinical trials often underrepresent diverse populations, leading to biased evidence bases. Less than 10% of participants in global oncology trials come from African or South Asian backgrounds [23], despite high disease burdens among people of these backgrounds. Future trial designs should require stratified recruitment by ethnicity and cultural background, with subgroup analyses published explicitly. Without such representation, medical evidence risks perpetuating therapeutic inequities.

## 6. Integration into Clinical Training and Practice

**A.**  **Curricular redesign.**

Medical curricula must integrate transcultural clinical medicine not as an elective, but as a required competency [24]. Case-based modules could focus on how salt-sensitive hypertension alters antihypertensive selection in African-origin patients, or how East Asian β-cell insufficiency demands earlier insulin initiation [25]. Teaching must move beyond abstract “cultural sensitivity” into specific, medically actionable knowledge.

**B.**  **Clinical communication models.**

Culturally responsive communication is not limited to language translation. Physicians must learn frameworks for eliciting patients’ explanatory models of disease—asking what they believe caused their condition, what treatments they expect, and how illness affects their daily life. Evidence shows that incorporating explanatory models reduces non-adherence and improves patient satisfaction across chronic conditions [26].

**C.**  **Multidisciplinary integration.**

Effective culturally tailored management of chronic disease requires team-based approaches. Endocrinologists, cardiologists, nephrologists, and oncologists must collaborate with cultural mediators, medical anthropologists, and clinical pharmacists. For example, embedding pharmacists trained in ethnopharmacology into diabetes clinics can reduce harmful herbal–drug interactions frequently seen in Latino and South Asian populations.

## 7. Structural and Policy-Level Considerations

**A.**  **Health system redesign.**

At the macro level, health systems must restructure to accommodate cultural variation. In dialysis centers, incorporating dietary counseling sensitive to staple foods can improve adherence to phosphorus and potassium restrictions. Oncology units should provide gender-concordant providers in cultures where modesty prevents participation in screening. These structural shifts have measurable impacts on adherence and outcomes.

**B.**  **Insurance and reimbursement policies.**

Reimbursement structures should incentivize culturally tailored interventions. Coverage for pharmacogenomic testing, bilingual dieticians, and community health worker integration could reduce long-term healthcare costs. Studies have demonstrated that community-based culturally tailored diabetes interventions are cost-effective and can lead to significant savings over the long term [27].

**C.**  **Global health collaborations.**

Chronic diseases transcend borders; therefore, cross-cultural management must be framed as a global health priority. WHO-led consortia could develop evidence-based culturally stratified clinical guidelines akin to infectious disease protocols, adapted by region. International registries collecting pharmacogenomic, metabolic, and adherence data across populations would provide the evidence base for such guidelines. Transplantation services, for instance, require structured accommodation of religious and cultural values to ensure equitable organ allocation [28].

## 8. Case Vignettes for Clinical Illustration

**Case 1: Cardiovascular Pharmacogenomics in East Asia.**

A 58-year-old Japanese man undergoes stent placement after acute coronary syndrome. Standard dual antiplatelet therapy with aspirin and clopidogrel is initiated. Within three months, stent thrombosis occurs. CYP2C19 testing reveals poor metabolizer status, common in East Asian populations [20]. Switching to prasugrel resolves recurrence. Had pharmacogenomic screening been culturally embedded into practice, morbidity could have been avoided.

**Case 2: Diabetes Adherence in Latino Populations.**

A 42-year-old Mexican-American woman presents with uncontrolled T2DM (HbA1c 11.2%). She reports adherence to “natural” treatments (nopal cactus) but inconsistent use of prescribed metformin. A culturally trained provider integrates her use of nopal into a broader dietary plan while emphasizing the pharmacological necessity of metformin. By negotiating rather than dismissing cultural beliefs, adherence improves and HbA1c drops to 7.6% in six months.

**Case 3: Dialysis in Middle Eastern Populations.**

A 65-year-old Saudi man with ESRD resists cadaveric transplantation, citing religious concerns. A culturally sensitive nephrologist collaborates with local religious leaders to provide theological support. Ultimately, the patient accepts a kidney from his nephew, achieving graft survival comparable to Western cohorts. This illustrates the integration of religious-cultural dialogue into medical practice.

## 9. Conclusions

Chronic disease management is not culturally neutral. From pharmacogenomic variation [20] to dietary adherence and health-seeking behaviors, cultural frameworks profoundly shape medical outcomes. The challenge for 21st-century medicine is to operationalize this knowledge into actionable clinical practice. Future directions include expanding genomic integration [29], mandating cultural heterogeneity in clinical trials [23], and reengineering health systems. to accommodate cultural variance.

Culturally responsive medicine must move beyond abstract empathy into structured clinical practice, supported by robust evidence bases. In doing so, clinicians can not only improve individual outcomes but also reduce global inequities in chronic disease management. This paradigm is not optional; it is essential to the evolution of modern medicine in an increasingly interconnected world.

## Data Availability

No new data were created or analyzed in this study.
